# DNA sensing of dendritic cells in cancer immunotherapy

**DOI:** 10.3389/fmolb.2024.1391046

**Published:** 2024-05-22

**Authors:** Wei Qian, Jun Ye, Sheng Xia

**Affiliations:** ^1^Department of Immunology, School of Medicine, Jiangsu University, Zhenjiang, Jiangsu, China; ^2^The Center for Translational Medicine, The Affiliated Taizhou People’s Hospital of Nanjing Medical University, Taizhou School of Clinical Medicine, Nanjing Medical University, Taizhou, Jiangsu, China

**Keywords:** dendritic cell, DNA sensor, cGAS, STING, TLR9, cancer immunotherapy

## Abstract

Dendritic cells (DCs) are involved in the initiation and maintenance of immune responses against malignant cells by recognizing conserved pathogen-associated molecular patterns (PAMPs) and damage-associated molecular patterns (DAMPs) through pattern recognition receptors (PRRs). According to recent studies, tumor cell-derived DNA molecules act as DAMPs and are recognized by DNA sensors in DCs. Once identified by sensors in DCs, these DNA molecules trigger multiple signaling cascades to promote various cytokines secretion, including type I IFN, and then to induce DCs mediated antitumor immunity. As one of the potential attractive strategies for cancer therapy, various agonists targeting DNA sensors are extensively explored including the combination with other cancer immunotherapies or the direct usage as major components of cancer vaccines. Moreover, this review highlights different mechanisms through which tumor-derived DNA initiates DCs activation and the mechanisms through which the tumor microenvironment regulates DNA sensing of DCs to promote tumor immune escape. The contributions of chemotherapy, radiotherapy, and checkpoint inhibitors in tumor therapy to the DNA sensing of DCs are also discussed. Finally, recent clinical progress in tumor therapy utilizing agonist-targeted DNA sensors is summarized. Indeed, understanding more about DNA sensing in DCs will help to understand more about tumor immunotherapy and improve the efficacy of DC-targeted treatment in cancer.

## Introduction

Dendritic cells (DCs) are a type of antigen-presenting cells (APCs) serving as a “bridge” between innate and adaptive immunity. They thus have a distinctive potential to trigger robust antitumor immunity in cancer. Based on its characteristics, human and mouse DCs are generally categorized as conventional DC1s (cDC1s), conventional DC2s (cDC2s), plasmacytoid DCs (pDCs), and monocyte-derived DCs (moDCs) (Wculek et al., 2020). cDC1s development rely on IRF8, ID2, and BATF3, which are transcription factors that selectively express the chemokine receptor XCR1 and the lectin receptor CLEC9A. cDC1s capture and transport tumor-associated antigens (TAAs) to tumor-draining lymph nodes and then efficiently cross-present TAAs through MHC-I to initiate tumor-specific CD8^+^ T cell responses (Böttcher and Reis E Sousa, 2018). Tumor-infiltrating cDC1s secrete CXCL10 to recruit CD8^+^ T cells and regulate cell functions by secreting T cell-associated cytokines (Spranger et al., 2017; Hubert et al., 2020). cDC2s require the transcription factors RELB, IRF4, and ZEB2 and are mainly involved in MHC-II-dependent antigen presentation in tumor-draining lymph nodes to initiate CD4^+^ T cells (Wculek et al., 2020). pDCs development depend on IRF8 and E2-2 expression and play a crucial role in antitumor immunity because of their robust capacity to produce IFN-I. Paradoxically, other studies showed that pDCs infiltration in the tumor microenvironment (TME) was associated with impaired IFN-I secretion, T cell response suppression, and Treg expansion, which ultimately leads to tumor immune escape (Reizis, 2019). Although MoDCs are mostly differentiated from monocytes in peripheral tissues during inflammation, they also have the similar ability to present antigens to CD8^+^ T cells (Wculek et al., 2020).

Chromosome instability is one of cancer hallmarks. It is characterized by chromosome segregation errors during mitosis, which results in micronuclei formation. Indeed, when genomic DNA (gDNA)-containing micronuclei ruptures, tumor DNA is exposed (Crasta et al., 2012). Along with the nuclear compartment, mitochondria is an additional source of genomic material, and mitochondrial dysfunction results in mitochondrial DNA (mtDNA) release. Furthermore, spontaneous tumor cell death and cancer therapy-induced immunogenic death increase the ability of immune cells to access these tumor-associated DNA (Ahmed and Tait, 2020). Innate immune cells use limited repertoires of germline-encoded pattern recognition receptors (PRRs) to identify conserved pathogen-associated molecular patterns (PAMPs) and damage-associated molecular patterns (DAMPs). Recently, DNA immunostimulatory properties have garnered substantial attention after the first sensor with DNA recognition was identified. The immunosensing of DNA has evolved to induce host immune responses in response to the invasion and exposure of exogenous nucleic acids (Hornung and Latz, 2010). Thus, DNA sensing is a pivotal player in antitumor immunity. Defects in the DNA sensing pathway are associated with tumor progression, and treatment using DNA sensor-targeting agonists can be a promising cancer therapeutic strategy (Woo et al., 2014; McWhirter and Jefferies, 2020).

In this review, we focus on the specific mechanisms in which DCs employ DNA sensors to recognize tumor-derived DNA and initiate an antitumor immune response. We also discuss how the tumor microenvironment (TME) inhibits DNA sensing of DCs to promote immune evasion, and the strategies to enhance DNA sensing in cancer immunotherapy. Finally, recent advances in DNA sensor agonists which suitable for cancer immunotherapy are also summarized.

### DNA sensors in DCs

**FIGURE 1:**
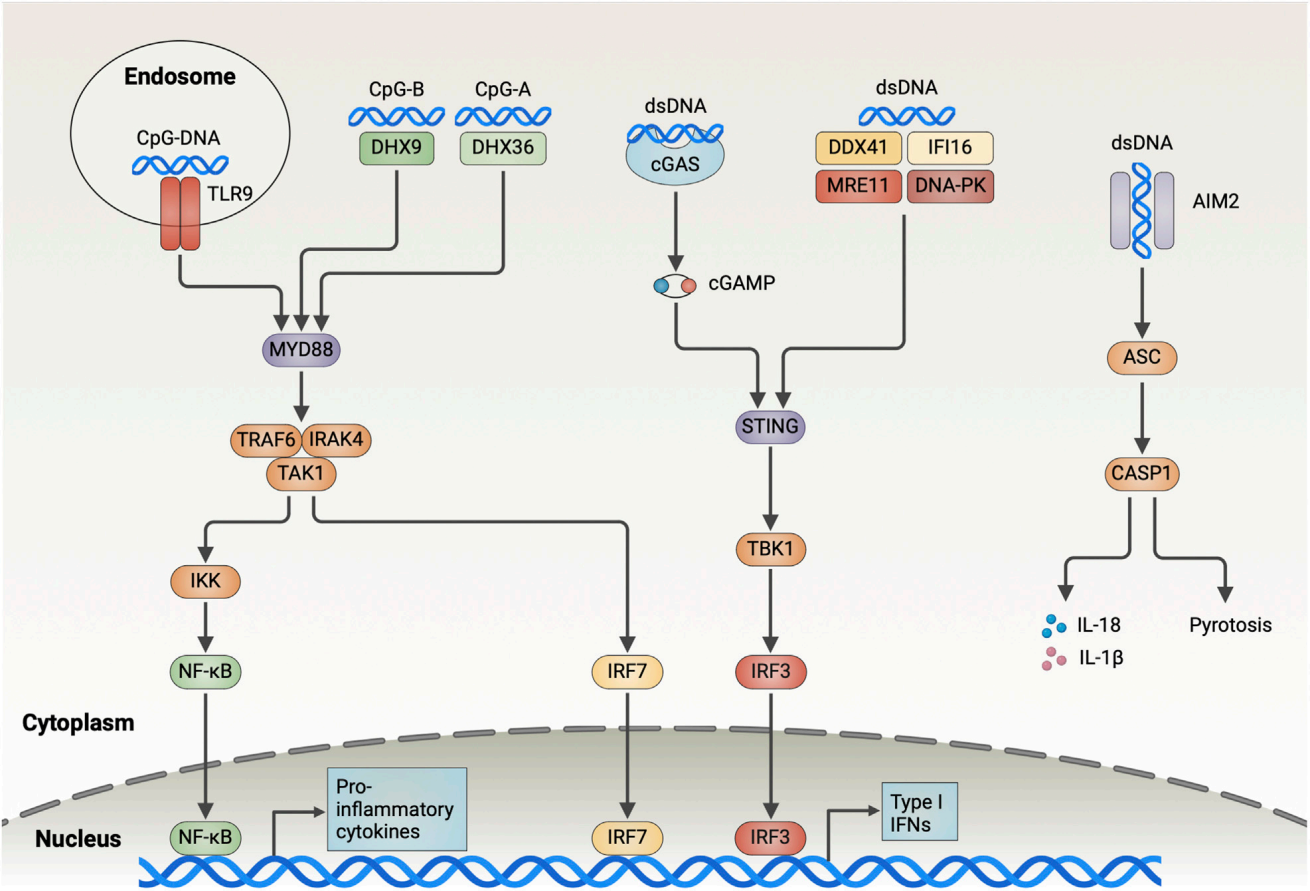
Overview of the DNA-Sensing Pathways in DCs. Endosomal TLR9 recognizes DNA containing CpG sequences and recruits IRAK4 and TRAF6 through MyD88, which contributes to the activation of TAK1. TAK1 further activates NF-κB and IRF7, which induces the expression of type I interferon and pro-inflammatory cytokines. DHX9 and DHX36 detect CpG-B and CpG-A in the cytoplasm, respectively, and trigger downstream signaling through MyD88. cGAS binds dsDNA in the cytoplasm in a sequence-independent manner and catalyzes the generation of cGAMP from ATP and GTP. Subsequently, cGAMP activates the endoplasmic reticulum-resident adaptor protein STING, triggering its dimerization and translocation to the Golgi, where it interacts with TBK1 to drive IRF3 activation and the type I IFN expression. DDX41, IFI16, MRE11, and DNA-PK bind cytoplasmic DNA and trigger downstream signaling through STING, albeit the detailed mechanisms remain unclear. AIM2 is responsible for detecting dsDNA in the cytoplasm and forming inflammasome by recruiting ASC and caspase-1. AIM2 inflammasome is involved in inducing pyroptosis and results in the release of mature IL-18 and IL-1β out of the cells (Created with BioRender.com).

In this section, we describe the DNA sensors identified in DCs. These sensors are localized in endosomes or the cytoplasm and have different mechanisms for sensing DNA (Figure 1).

### Toll-like receptor 9

Toll-like receptor 9 (TLR9) is the first PRR identified with a DNA-recognition function. It detects unmethylated CpG-containing single-stranded DNA in endosomes. The multi-transmembrane protein Unc93B1 transports TLR9 from the endoplasmic reticulum (ER) to the endolysosome and proteolytically cleaves its ectodomain to activation (Fukui et al., 2018). The TLR9 conformation changes following ligand binding, thereby leading to myeloid differentiation primary response protein 88 (MyD88) and IL-1R-associated kinase (IRAK)- 4 being recruited by the Toll/IL-1 receptor (TIR) domain, which results in the assembly of MyD88–IRAK4 complex. This complex then interacts with IRAK-2 or IRAK-1 (Lin et al., 2010). Next, the activated TNF receptor-associated factor 6 (TRAF6) recruits TGF-β-activated kinase 1 (TAK1), which subsequently triggers IκB kinase (IKK) complex activation and NF-κB nuclear translocation. Additionally, TAK1 activates mitogen-activated protein kinases, thus stimulating nuclear translocation of activator protein 1 (AP1). NF-κB and AP1 transcription induces the expression of pro-inflammatory cytokines, including IL-12 and TNF-α. Moreover, MyD88 initiates IFN-I expression through transcriptional regulation of the IFN regulatory factor 7 (IRF7) (Blasius and Beutler, 2010).

TLR9 is mainly located in the endolysosomal compartment. Therefore, tumor cell-derived CpG DNA, which is ingested through endocytosis, mediates TLR9 activation in tumor-infiltrating DCs. So, tumor-released DNA induces intratumoral DCs accumulation, augments antigen uptake, and promotes DCs maturation in a TLR9-dependent manner. Subsequently, DCs migrate into lymph nodes, thereby activating tumor-specific cytotoxic T lymphocytes (CTLs) (Kang et al., 2019). Notably, when TLR9 is absent in bone marrow-derived dendritic cells (BMDCs), the IFN-I response is eliminated after CpG-ODN stimulation.

### cGAS and STING

The cyclic GMP–AMP synthase (cGAS), a nucleotidyltransferase family member, binds to cytoplasmic dsDNA in a sequence-independent manner (Sun et al., 2013). The conformation of activated cGAS changes in which it catalyzes cGAMP generation from ATP and GTP, and activates the stimulator of interferon genes (STING). Activated STING, an ER membrane protein, is oligomerized to form higher-order oligomers. It is then translocated from the ER to the Golgi apparatus through the ER–Golgi intermediate compartment. Subsequently, STING recruits TBK1 via the carboxyl terminus and induces IRF3 recruitment. Later, when phosphorylated IRF3 is dimerized, it translocates to the nucleus, thereby inducing IFN-I expression (Decout et al., 2021). Additionally, STING-mediated IKK activation leads to NF-κB release into the nucleus, which triggers the expression of inflammatory cytokines such as TNF, IL-1β, and IL-6 (Chen et al., 2016).

An analysis of immunogenic tumors in mice revealed that tumor-infiltrating DCs recognize tumor-derived dsDNA via cGAS, and the activation of STING-IFN-I signaling pathway leads to DC-initiated antitumor T-cell responses (Deng et al., 2014; Woo et al., 2014; Spranger et al., 2017). However, the precise mechanism through which tumor DNA escapes from endolysosomes to the cytoplasm following DC-mediated phagocytosis to activate cGAS remains elusive. On co-culturing tumor cells with BMDCs *in vitro*, Schadt et al. observed that cGAS silencing in DCs did not affect IFN-I secretion (Schadt et al., 2019). Furthermore, in lymphoma and melanoma tumor models, cGAS-deficient mice exhibited tumor growth similar to that of WT mice (Marcus et al., 2018). This suggests that the DNA sensor cGAS plays a redundant role in tumor recognition by DCs. Of note, tumor cells also express cGAS and spontaneously produce a low IFN-I level. Abnormal cytoplasmic dsDNA in tumor cells leads to cGAS activation and cGAMP production. The produced cGAMP is then translocated to DCs where it activates STING (Schadt et al., 2019). In one study, IFN-I was absent in co-cultures of cGAS-deficient CT26 tumor cells with wild-type BMDCs, compared with WT tumor cells (Schadt et al., 2019). These results revealed another mechanism of DNA sensing of DCs, in which tumor cell-derived cGAMP is recognized via STING. However, cGAMP exported by tumor cells into the TME is cleared by extracellular ENPP1, a cGAMP protein hydrolase (Carozza et al., 2020). It also showed that cGAMP translocation mainly relied on the vector SLC19A1 as well as gap junctions, a cell–cell communication mechanism (Luteijn et al., 2019; Ritchie et al., 2019; Schadt et al., 2019).

### PYHIN family proteins: AIM2 and IFI16

The cytosolic DNA sensor AIM2 has an N-terminal pyrin domain and a C-terminal HIN-200 domain. The HIN-200 domain interacts with dsDNA in a sequence-independent manner, subsequently recruiting apoptosis-associated speck-like protein containing CARD (ASC) and pro-caspase-1 through the pyrin domain to form a macromolecular complex called the AIM2 inflammasome. This inflammasome formation process activates caspase-1, which in turn cleaves pro-IL-1β and pro-IL-18, and activates the pore-forming protein gasdermin D (GSDMD). Next, GSDMD activation induces pyrotosis, an inflammatory form of cell death, and causes massive release of mature forms of IL-1β and IL-18 outside the cells (Man et al., 2016).

AIM2 expression in tumor-associated DCs correlates with human melanoma tumor progression. AIM2 silencing promotes tumor DNA sensing by BMDCs, as evidenced by augmented STING signaling (Fukuda et al., 2021). The AIM2 inflammasome manipulates DNA sensing of DCs through several mechanisms. The activated ASC protein interacts with STING and disrupts the STING–downstream TBK1 interaction, thereby reducing IFN-I production (Yan et al., 2018). Activated caspase-1 can bind and cleave cGAS through its p20 structural domain (Wang Y. et al., 2017). The membrane pores induced by gasdermin D, a pore-forming protein, lead to intracellular K^+^ efflux, thereby inhibiting cGAS-dependent IFN-β responses while inducing pyroptosis (Banerjee et al., 2018). Furthermore, in pDCs, AIM2 inflammasome activation inhibits MyD88-IRF7-mediated IFN-I signaling by upregulating SOCS1 expression, which is a crucial pathway through TLR9 sensing DNA (Yu et al., 2018). Preventing AIM2 activation in DCs positively affects DC-mediated DNA sensing. Further studies unveiled that the AIM2-deficient DC vaccine, as an adjuvant, recruits more antigen-specific CD8^+^ T cells by generating CXCL10, and improves the efficacy of the combination of adoptive T-cell therapy and anti-PD-1 antibodies against melanoma. Furthermore, AIM2 deficiency decreases IL-1β and IL-18 production, which prevents regulatory T-cell infiltration (Fukuda et al., 2021). Notably, Jiang et al. reported that tumor-associated APCs highly express GSDMD, a key pore-forming protein that induces pyroptosis following AIM2 activation. Single-cell RNA-seq analysis demonstrated that GSDMD deletion in APCs augmented the expression of cGAS-dependent interferon-stimulated genes, thereby enhancing antigen presentation and facilitating CD8^+^ T-cell-mediated antitumor immunity (Jiang et al., 2022). Collectively, these findings underscore the possible therapeutic significance of blocking the AIM2 inflammasome to potentiate DNA sensing of DCs to promote antitumor immune responses.

As PYHIN protein family members, human IFI16 and its mouse immediate homolog, IFI204, serve as cytoplasmic DNA sensors that induce IFN-β production by recruiting STING (Unterholzner et al., 2010). In cGAS-deficient DCs, IFI204 knockdown abrogated inducible STING phosphorylation following cytosolic DNA stimulation, thereby underlining that IFI204 plays a crucial role in STING activation (Hu et al., 2021). Additionally, the deSUMOization of IFI204, a protein post-translational modification, facilitated the IFI204–STING interaction and augmented STING-dependent DNA sensing by DCs (Hu et al., 2021).

### DExD/H-box helicases

Another protein family involved in DNA sensing is RNA helicases, which have two subsets: DEAH-box helicases (DHX) and DEAD-box helicases (DDX). In a study on pDCs, DHX9 recognized CpG-B and induced IFN-I through the MyD88-IRF7 pathway, whereas DHX36 recognized CpG-A and induced TNF-α and IL-6 through the MyD88-NF-κB pathway (Kim et al., 2010). However, Zhang et al. reported that mDCs with DHX9 or DHX36 knockdown exhibited normal cytokine responses to B-form DNA (Zhang et al., 2011). Furthermore, another study reported that DHX9 acts as a transcriptional co-activator in APCs, which augments NF-κB activation in a DNA sensing-independent manner (Ng et al., 2018). Collectively, these results highlight the redundant role of DHX9 and DHX36 in DNA sensing, which remains unexplored.

DDX41 binds DNA and STING through the DEADc structural domain and triggers IFN-I production through the TBK1-IRF3 signaling pathway (Zhang et al., 2011). According to the research of Singh et al., 2022, DDX41 exhibits DNA unwinding and single-stranded annealing activity. DDX41 regulates cGAS activation by modulating cytoplasmic dsDNA and ssDNA homeostasis. Thus, whether DDX41 serves as a sensor for direct DNA sensing or as a molecule with DNA-binding functions involved in regulating DNA sensing remains unclear.

### DNA-PK

The DNA-dependent protein kinase (DNA-PK) is another major component involved in cellular response to DNA damage and cytoplasmic DNA sensing. It comprises three subunits, namely, ku70, ku80, and the catalytic subunit DNA-PKcs. According to the study of Ferguson et al., DNA-PK binds to cytoplasmic DNA and induces IRF3-dependent innate immune responses (Ferguson et al., 2012). Ma et al. reported that DNA-PKcs is involved in regulating TLR9-mediated DNA sensing. DNA-PK loss severely abrogated the IFN response to CpG-ODN in BMDCs, but the detailed mechanisms remain unclear (Ma C. et al., 2013; 2015). Thus, this study demonstrated the role of DNA-PK in DNA sensing of DCs. In THP-1 cells, however, DNA-PK phosphorylated cGAS, which suppressed cGAMP generation (Sun et al., 2020). Another study also reported the STING-independent DNA sensing function of DNA-PK in human cells, with the heat shock proteins (HSPs) HSPA8/HSC70 acting as downstream targets (Burleigh et al., 2020). These aforementioned findings further indicate the complexity of the DNA-PK function in DC-mediated DNA sensing. Therefore, DNA-PK signaling and the signal crosstalk between DNA-PK and other DNA sensors must be clarified. Additionally, preclinical studies have reported that human moDCs treated with the DNA-PK inhibitor NU7441 exhibited increased MHC-I molecule expression, whereas PD-L1 and PD-L2 expression was downregulated (Guo et al., 2019). This suggests a strong potential strategy by using DNA-PK inhibitors as drug candidates in DC vaccine preparation for cancer treatment.

### MRE11

The MRE11-RAD50-NBS1 (MRN) complex, an ATP-dependent nuclease, cleaves both unbound and obstructed DNA termini to repair DNA through end joining or homologous recombination (Hopfner, 2023). MRE11 degrades nascent mtDNA replication forks to induce mtDNA instability, which then triggers cGAS-activated accumulation of cytoplasmic DNA substrates (Luzwick et al., 2021). MRE11 thus functions as a nuclease involved in mitochondria-dependent cGAS activation. Furthermore, Kondo et al. revealed that MRE11 is a pivotal player in the recognition of cytoplasmic dsDNA and initiation of STING-dependent signaling, which is not related to its nuclease activity. shRNA-mediated MRE11 knockdown in BMDCs abrogated IFN-I induction in response to DNA (Kondo et al., 2013). In a recent study, when MRN complexes bind to nucleosome fragments, cGAS is released from histones, which enables cGAS mobilization and activation by dsDNA (Cho et al., 2024). So, these results suggest that MRE11 is involved in regulating STING-mediated DNA sensing through binding to broken DNA.

### TME and DNA sensing in DCs

Because of their heightened metabolic activity, tumor cells usually generate substantial amounts of metabolic byproducts. If these waste products accumulated in the TME, the function of tumor-infiltrating immune cells would be negatively regulated. Here, we discuss how severe TME alters DNA sensing in DCs.

### Hypoxic responses

Hypoxia is observed in the TME of most solid tumors, as oxygen diffusion limitations exceed the distance between tumor cells and the vascular system (He et al., 2022). Hypoxia in the TME increases HIF-1α levels in DCs. Indeed, it activates glycolytic HIF target genes encoding enzymes, such as hexokinase 2 (HK2) and pyruvate kinase M2 (PKM2), ultimately leading to a metabolic switch from mitochondrial metabolism to glycolysis, which represents a metabolic strategy for meeting the energy requirements of DCs(Jantsch et al., 2008). Glycolytic reprogramming stimulates DC maturation, migration, and T-cell priming (Guak et al., 2018; Liu et al., 2019). In a recent study, enhanced glycolysis augmented glycolytic ATP production in tumor-infiltrating DCs, which drove STING signaling to facilitate DC-mediated antitumor immune responses (Hu Z. et al., 2023). Additionally, TLR9-induced IFN-I secretion by human pDCs was impaired following the administration of 2-deoxy-D-glucose (2-DG), a glycolysis inhibitor (Fekete et al., 2018). According to these results, elevated intrinsic glycolysis augments DNA sensing of DCs. However, the mechanism in which glycolytic ATP activates DNA sensors remains unclear. Interestingly, the flow of glycolytic and pentose phosphate pathways in DCs increased rapidly following stimulation with CpG, cGAMP, and tumor DNA. By increasing the intracellular succinate concentration and driving the production of mitochondrial reactive oxygen species, DCs stabilize HIF-1α to induce glycolysis (Gomes et al., 2021). This indicates that glycolytic metabolic reprogramming occurs in DCs after DNA sensing. Taken together, DNA sensing by DCs accelerates HIF-1α-mediated glycolysis, and glycolysis further improves this DNA sensing, which thus establishes a positive feedback loop. However, STING was recently reported to restrict aerobic glycolysis by targeting hexokinase II (HK2) and blocking its hexokinase activity, which is independent of its innate immune recognition function (Zhang et al., 2023). After all, the TME induces hypoxic stress in DCs. Under such hypoxic stress, DCs exhibit altered metabolic activities and can recognize tumor DNA more efficiently.

### Lactate

Lactate is primarily produced through the “Warburg effect” of tumor cells in the TME. Tumor cells restore metabolism, increase glucose uptake, and ferment glucose to lactate through glycolysis, even under well-oxygenated conditions (Liberti and Locasale, 2016). Extracellular lactate interacts with cells via G protein-coupled receptor 81 (GPR81) on the cell surface or is directly transported into cells through the monocarboxylate transporter (MCT) on the cell surface. MCT-1 is the primary lactate importer, whereas MCT-4 is one key lactate exporter (Caslin et al., 2021). Tumor cells actively secrete substantial amounts of lactate into the TME through MCT-4, and the secreted lactate activates GPR81 on tumor cells. This autocrine mechanism is associated with tumor growth, metastasis, and immune evasion (Roland et al., 2014; Feng et al., 2017).

Notably, lactate in the TME also activates the GPR81 receptor on DCs. In a study on pDCs, Ca2^+^ signaling following TLR9 activation induced a downstream CAMKII signaling cascade at physiological calcium concentrations, which resulted in IFN-I release (Raychaudhuri et al., 2020). Following lactate-mediated activation of GPR81 on pDCs, Gβγ subunit-dependent intracellular Ca2^+^ mobilization is induced. When the cytoplasmic Ca2^+^ level exceeds its physiological concentrations, Ca2^+^ activates CALN signaling rather than CAMKII signaling, which inhibits TLR9 activation (Raychaudhuri et al., 2019). Moreover, CHBA, a GPR81-specific agonist, eliminates the immunostimulatory response of DCs to exogenous DNA and weakens their antigen-presenting function (Brown et al., 2020). This suggests that lactate activates the GPR81-Ca2^+^-CALN signaling pathway and inhibits DNA sensing of DCs. Of note, DCs express MCT-1 that mediate the cytoplasmic import of lactate into the TME. After lactate enters the cell, lactate and its associated H^+^ ions accumulate and serve as negative feedback regulators controlling glycolytic ATP production, which is essential for DNA sensing in DCs (Raychaudhuri et al., 2019). This indicates that lactate inhibits DNA sensing of DCs by influencing the cellular metabolism required for DCs activation. Additionally, lactate accumulation in pDCs enhances tryptophan metabolism and kynurenine production, which then leads to the expansion of FoxP3^+^CD4^+^ regulatory T cells and promotes TME immunosuppression (Raychaudhuri et al., 2019). In summary, effective lactate metabolism is critical for DCs to perform their physiological function in the TME. Lactate accumulation disrupts acid–base homeostasis and inhibits DNA sensing of DCs, thus resulting in tumor immune escape.

### Reactive oxygen species

As a byproduct of aerobic respiration, DC-generated reactive oxygen species (ROS) in the TME are chiefly derived from increased NADPH oxidase (NOX) activity, fatty acid β-oxidation, and inefficient electron conduction in the mitochondrial electron transport chain (Giovanelli et al., 2019). In addition, DCs phagocytose ROS-containing microvesicles (MVs) secreted by tumor cells and thus uptake exogenous ROS (Battisti et al., 2017). Numerous studies have linked ROS to DNA sensing of DCs. On evaluating the ROS level in BMDCs, a study found that ROS^lo^ DCs were highly responsive to TLR stimulation, whereas ROS^hi^ DCs exhibited a low response (Sheng et al., 2010). This indicates that different ROS levels exert varying influences on DNA sensing functions. First, NOX mediates TLR9 translocation from the ER to endosomes, which is essential for TLR9 to play its DNA-sensing function (Müller-Calleja et al., 2023). Upon TLR activation, NOX2 is recruited to the phagosomes of DCs to mediate sustained production of low ROS levels. ROS inhibits acid hydrolase activity by inducing endolysosomal alkalinization, which then prevents nucleic acid degradation in endosomes and promotes DNA sensing by TLR9 (Savina et al., 2006). Interestingly, DC-derived ROS also trigger SENP3 accumulation and the SENP3–IFI204 interaction, thus catalyzing IFI204 deSUMOylation and boosting STING signaling activation (Hu et al., 2021). These aforementioned results suggest that low ROS levels can promote DNA sensing in DCs.

Tumor-infiltrating DCs usually have higher ROS levels because of TME’s complexity (Hu et al., 2021). High ROS levels induce DNA damage, lipid peroxidation, and protein denaturation, which have more complex effects on DNA sensing of DCs. Guanine oxidation to 8-hydroxyguanine (8-OHG) is a characteristic of oxidative damage to DNA. 8-OHG potentiates STING-dependent DNA sensing via resisting cytoplasmic 3′ repair exonuclease 1 (TREX1)-mediated degradation (Gehrke et al., 2013; Pazmandi et al., 2014; Fang et al., 2021). Furthermore, ROS-induced lipid peroxidation alters the permeability of endolysosomal and mitochondrial membranes, thereby resulting in DNA leakage and triggering cytoplasmic DNA sensing (Dingjan et al., 2016; Cheng A. N. et al., 2020). Recent studies have also unveiled the inhibitory effect of ROS on STING. In macrophages, ROS directly oxidize cysteine 147 on STING, thus inhibiting STING polymerization and activation of downstream signaling events (Tao et al., 2020). Additionally, inactivation of glutathione peroxidase 4 augmented lipid peroxidation and subsequent STING carbonylation at C88, which impeded the transport of STING from the ER to the Golgi complex (Jia et al., 2020). In contrast to the DCs phagocytic pathway adapted to antigen presentation, macrophages terminate invading pathogens through oxidative bursts of ROS. Abundant ROS production simultaneously hinders STING-mediated DNA sensing. Furthermore, ROS is involved in the induction of various cell death forms, such as reticulocyte death, ferroptosis, and oxepitosis (Galluzzi et al., 2018; Holze et al., 2018).

### ER stress

Protein folding, modification, and secretion must be executed efficiently for DCs to respond and adapt to the activation of innate immune sensors. Despite these processes being tightly regulated, various extrinsic and intrinsic factors can disrupt ER’s protein folding capacity, thus leading to ER stress, which is characterized by abnormal accumulated misfolded proteins. Subsequently, multiple events occur to facilitate the restoration of ER homeostasis, including the unfolded protein response (UPR), ER-associated degradation (ERAD), and autophagy (Chen and Cubillos-Ruiz, 2021).

Three ER transmembrane stress sensors, namely, IRE1α, PERK, and ATF6, cooperatively activate the UPR. The IRE1α-XBP1 arm of the UPR plays an essential role in DC homeostasis. When XBP1 in DCs is specifically deleted, lung cDC1s are lost due to apoptosis (Tavernier et al., 2017). XBP1 loss also impairs the cDC1s phenotype and antigen presentation function, which results from the IRE1α-dependent degradation of mRNAs encoding MHC-I molecules (Osorio et al., 2014). Additionally, the IRE1α-XBP1 arm primarily mediates prostaglandin synthesis in DCs by promoting Cox-2 and PGES-1 expression (Chopra et al., 2019). However, ROS is accumulated within dysfunctional tumor-associated DCs, which triggers sustained activation of ER stress and UPR by producing lipid peroxidation byproducts that modify ER-resident proteins (Cubillos-Ruiz et al., 2015). The IRE1α-XBP1 arm modulates lipid metabolism, promotes triglyceride biosynthesis, and induces abnormal lipid accumulation, thus inhibiting the antigen-presenting function of DCs and antitumor T-cell responses (Cubillos-Ruiz et al., 2015). IRE1a-XBP1 signaling is also involved in regulating DNA sensing of DCs. Chaudhary et al. reported that IRE1α-XBP1 signaling activation suppresses IFN-I production by TLR9-activated pDCs. XBP1 activation induces the expression of phosphoglycerate dehydrogenase and relinks glycolysis to serine biosynthesis. With this process, pyruvate’s access to the tricarboxylic acid cycle is reduced and mitochondrial ATP (mATP) generation is impaired. mATP is essential for TLR9 recognition in pDCs (Chaudhary et al., 2022). In addition, IRE1α-XBP1 signaling is involved in the lysosomal degradation of STING at the resting state (Pokatayev et al., 2020). Thus, blocking of IRE1α-XBP1 signaling facilitates DNA sensing of DCs. However, In macrophages, XBP1 increases cytoplasmic mtDNA release by inhibiting BNIP3-mediated activation of mitophagy, which then promotes cytoplasmic cGAS-STING signaling (Wang Q. et al., 2022). A study from our collaborators unveiled that IRE1α-XBP1 pathway inhibition or activation through pharmacological and genetic methods caused no alteration in STING protein expression levels (Ji et al., 2023). Therefore, additional studies are warranted to clarify the relationship between IRE1α-XBP1 signaling and cGAS-STING signaling.

At the steady state, DCs maintain protein folding homeostasis by activating PERK-mediated eIF2α phosphorylation. PERK inactivation increases overall protein synthesis and regulates IFN-β expression, while impairing LPS-stimulated DCs migration (Mendes et al., 2021). Additionally, PERK activation-induced transcription factor CHOP participates in IL-23 expression in DCs by binding to the IL-23 promoter (Goodall et al., 2010). A recent study revealed a non-classical cGAS-STING-PERK pathway. Following cGAMP binding, STING interacts with and induces PERK activation in the ER, independent of the UPR. When this pathway is activated, mRNA translation is significantly inhibited at the overall cellular level, but the synthesis of inflammatory- and survival-preferred proteins is specifically promoted (Zhang et al., 2022). Furthermore, PERK is involved in stress-mediated ER autophagy, which coordinates the interferon response by translocating the ER-resident STING to autophagosomes (Moretti et al., 2017). According to the aforementioned results, PERK may be specifically involved in maintaining DC homeostasis and function after DNA sensing.

The relationship between ATF6 and DCs remains poorly understood. ER stress-induced ATF6 activation has been suggested to enhance the pro-inflammatory properties of liver kupffer cells (KCs) in response to TLR stimulation. ATF6 siRNA-treated KCs produce lower levels of TNF-α and IL-6 but higher levels of IL-10 (Rao et al., 2014). Additionally, chronic ER stress causes the release of ATF6-dependent mtDNA, which then triggers cGAS-STING-dependent IFN-I responses (Rösing et al., 2024). This indicates that ATF6 activation positively affects DNA sensing of DCs.

Being a conserved quality control mechanism, ERAD is responsible for the removal of misfolded proteins from the ER. Our current findings indicate that ERAD is essential for DCs homeostasis. ERAD dysfunction induces cDC1s loss in different mouse organs, and the related mechanisms are being investigated. The relationship between ERAD and STING-mediated innate immunity has been elaborated in recent studies. ERAD negatively regulates STING-mediated DNA sensing in macrophages under basal conditions. For this purpose, it ubiquitinates and targets nascent STING proteins for proteasomal degradation (Ji et al., 2023). In addition, the E3 ubiquitin ligases RNF5 and TRIM30α serve as negative feedback regulators involved in ubiquitination degradation following STING activation (Wang et al., 2015; Yang et al., 2022). This suggests that ERAD regulates STING abundance in the basal state and STING activity in the activated state.

### Tumor immunotherapy and DNA sensing in DCs

**FIGURE 2:**
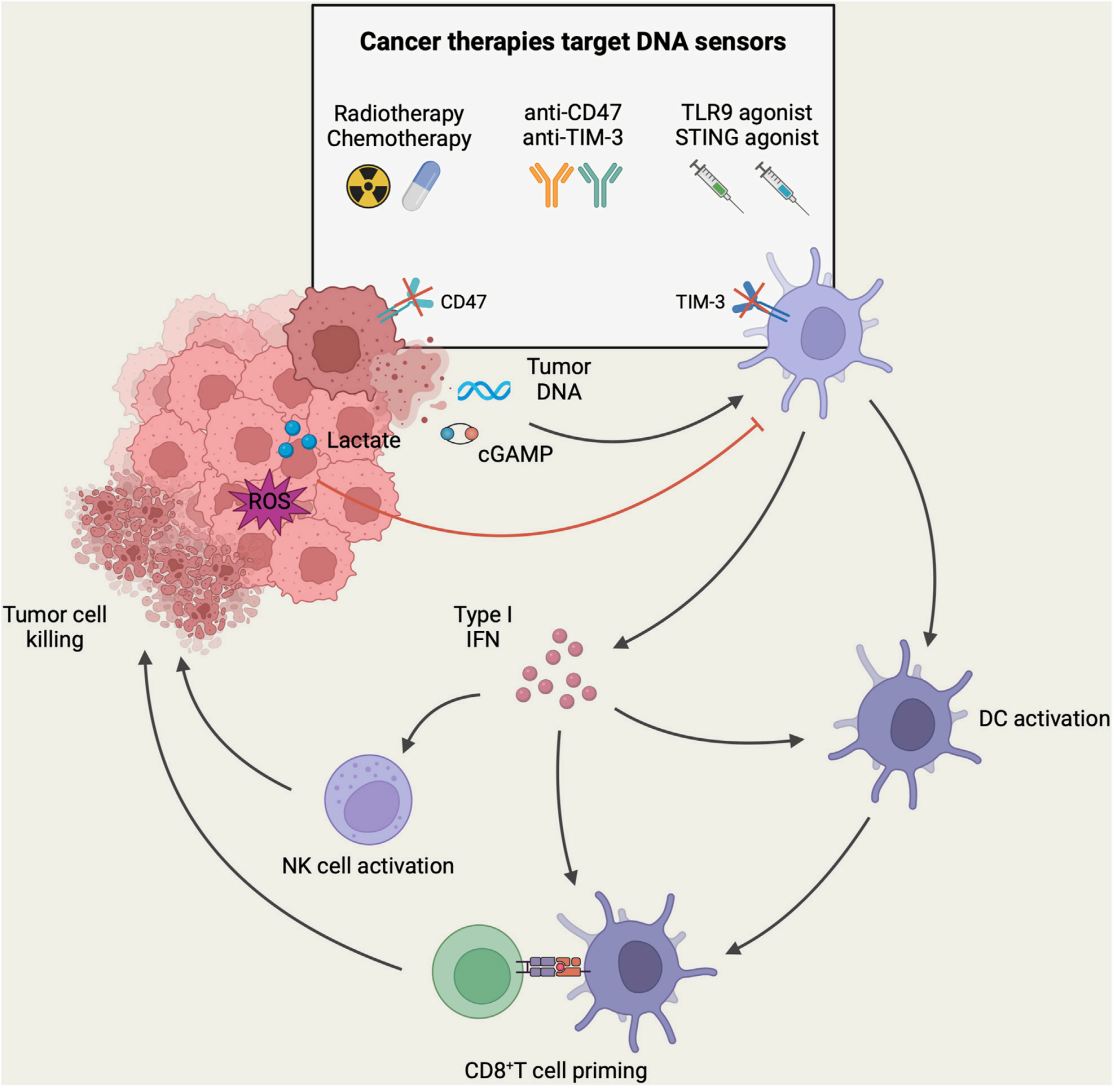
DNA-sensing models of DCs in the context of antitumor immunity. In TME, DNA and cGAMP derived from tumor cells can be recognized by DNA sensors in DCs, resulting in the activation of tumor-infiltrating DCs and the subsequent induction of type-I IFN. These activated DCs migrate to tumor-draining lymph nodes, where they present tumor antigens to CD8^+^ T-cells. Subsequently, CD8^+^ T-cells are recruited to the tumor site for mediating cytotoxicity against tumor cells. In addition, the secretion of type-I IFN facilitates the recruitment and activation of NK cells for direct tumor killing. However, high levels of lactate and ROS in TME can inhibit DNA sensing of DCs and mediate tumor immune escape. DNA sensing of DCs can be targeted to enhance anti-tumor immune response in tumor immunotherapy. Currently, different tumor immunotherapy strategies have been designed to target the DNA sensing of DCs to enhance the host anti-tumor immune response. Herein, radiotherapy and chemotherapy induce immunogenic death of tumor cells to release more tumor-associated DNA. Immune checkpoint therapies such as anti-CD47 and anti-TIM-3 antibodies were employed to enhance the phagocytosis of DCs to increase the uptake of tumor DNA. Furthermore, intratumoral injection of agonists targeting TLR9 or STING could directly activate DNA sensors in tumor-associated DCs, leading to DC activation and the initiation of an anti-tumor immune response (Created with BioRender.com).

Currently, tumor immunotherapies used in clinics can affect or even depend on DNA sensing of DCs. In this section, we discuss how these therapeutic interventions enhance DNA sensing in DCs (Figure 2).

### Genotoxic cancer therapy

Genotoxic cancer therapies kill tumor cells by inducing DNA damage. Chemotherapy and radiotherapy (RT) are the most commonly applied genotoxic therapies via triggering cell cycle arrest or cell death by forming double-stranded breaks, single-stranded breaks, and interstrand cross-links (Larsen et al., 2022). Chemotherapy and radiotherapy have been linked to the activation of DNA sensing, interferon secretion, and initiation of CD8^+^ T cell responses (Deng et al., 2014; Barlow et al., 2016). Genotoxic cancer therapies are known to manipulate DNA sensing of DCs through two key mechanisms, namely, induction of immunogenic cell death (ICD) and activation of intrinsic DNA sensing in tumor cells.

It is well known that radiotherapies and chemotherapies can induce ICD in tumor cells. Dying tumor cells generate new antigenic epitopes and release DAMPs, which include tumor DNA. These DAMPs can be recognized by DNA sensors expressed in DCs (Ahmed and Tait, 2020). ATP is another crucial DAMP that recruits CD11c^+^CD11b^+^Ly6C^high^ DCs to tumors in a CCL2/CCR2-dependent manner and stimulates local differentiation of this functional APC population (Ma Y. et al., 2013; 2014). Additionally, tumor cells express calreticulin and HSPs after chemotherapy, and the exposure of these “eat-me” signals promotes cellular uptake of DCs(Obeid et al., 2007). Similarly, cancer cell-released HMGB1 mediates extracellular DNA uptake by CD103^+^ DCs via forming HMGB1–DNA complexes (de Mingo Pulido et al., 2021). Altogether, immunogenic death of tumor cells promotes DNA sensing of DCs by increasing the opportunities for contact between DCs and DNA in the TME and by stimulating phagocytosis of DNA by DCs.

The progression of the cell cycle during mitosis after exogenous DNA damage leads to micronuclei formation. These micronuclei consist of chromosomal DNA surrounded by their nuclear membrane. When the micronuclear envelope is disrupted, gDNA leaks into the cytoplasm (Harding et al., 2017; Mackenzie et al., 2017). Moreover, DNA damage activates the pro-apoptotic protein BAK/BAX and leads to permeabilization of the mitochondrial outer membrane. When mitochondrial permeability changes, mtDNA is exposed to the cytoplasm (McArthur et al., 2018). Thus, genotoxic cancer therapy-induced extensive DNA damage results in massive DNA accumulation in the cytoplasm. Then, these ectopic DNAs trigger intrinsic cGAS-STING activation in tumor cells, thus leading to IFN-I release (Yang et al., 2021). According to recent studies, activation of intrinsic DNA sensing in tumor cells is essential for DC-mediated DNA sensing. First, RT-stimulated IFN-I secretion by tumor cells is required for recruiting and activating Batf3-dependent DCs, which is essential for initiating tumor-specific CD8^+^ T cell response. Furthermore, tumor cells with a deficient cGAS-STING-IFN pathway cannot initiate RT-induced antitumor immunity (Vanpouille-Box et al., 2017). This indicates that RT-induced IFN-I production enhances tumor immunogenicity. Remarkably, RT induces increased cGAMP export from tumor cells to the extracellular compartment, thereby directly activating STING in DCs, and extracellular cGAMP levels correlate with the RT efficacy (Carozza et al., 2020). Thus, intrinsic DNA sensing in tumors is important for RT-induced antitumor immune response.

However, some studies showed that tumor cells fail to effectively produce IFN-I in genotoxic cancer therapies. Restricted intrinsic DNA sensing in tumors suggests the presence of immunosuppressive mechanisms. During RT, tumor cells activate caspase-activated DNAase, which limits premature mitotic progression by reversibly increasing the number of genome-wide DNA breaks and ultimately avoids gDNA leakage (Larsen et al., 2022). Radiation doses of greater than 12–18 Gy induce DNA exonuclease Trex1, which can degrade DNA accumulating in the cytosol following radiation exposure (Vanpouille-Box et al., 2017). Furthermore, irradiated tumor cells hijack apoptotic caspase so as to inhibit mtDNA-induced STING-dependent IFN-I production (White et al., 2014; Han et al., 2020). Thus, cancer cell-induced apoptotic caspase cascades mediate silencing of immunorecognition of mitochondrial apoptosis. Unfortunately, activation of intrinsic DNA sensing in tumors sometimes does not mean increased antitumor immunity. A more recent study demonstrated that intracellular cGAS-STING-activated cancer cells can resist chemotherapy drug-induced stress by hijacking evolutionarily conserved NF-κB inflammatory signaling (Lv et al., 2023). Moreover, IRF3, a downstream transcription factor of the cGAS-STING pathway, directly augmented PD-L1 transcription in hepatocellular carcinoma cells following radiation exposure. PD-L1 binds and stabilizes mRNA levels of NBS1, BRCA1, and other genes associated with the DNA damage response, thereby increasing cellular resistance against DNA damage (Tu et al., 2019; Du S.-S. et al., 2022).

### Immune checkpoint blockade

The discovery of immune checkpoints and the development of immune checkpoint inhibitors (ICIs) have revolutionized the tumor immunology field, thus increasing the treatment opportunities for some cancer patients. Most immune checkpoint therapies function primarily by stimulating adaptive immunity, particularly through promoting T-cell response, such as the usage of CTLA-4 and PD-1/PD-L1 monoclonal antibodies. As innate immune cells, DCs also express innate immune checkpoints. DCs use these checkpoints to detect and eliminate tumor cells through phagocytosis. Phagocytic checkpoints in cancer immunotherapy have been previously reviewed (Liu et al., 2023). Indeed, targeting phagocytic checkpoints for increasing the phagocytic function of DCs may mean increased DNA sensing and recent studies have predominantly focused on CD47 and TIM-3.

CD47 is a protein with the function of transmitting the “don't eat me” signal. It is usually overexpressed on the tumor cell surface and mediates tumor cell immune evasion by inhibiting DC-mediated phagocytosis through signal regulatory protein α (SIRPα) (Cheng-En et al., 2022). When CD47 binds to the inhibitory receptor SIRPα, phosphorylation of the immunoreceptor tyrosine-based inhibitory motifs (ITIMs) of SIRPα is promoted, which recruits SHP-1 and SHP-2 to dephosphorylate the motor protein myosin IIA and thus inhibit phagocytosis (Logtenberg et al., 2020). Furthermore, high CD47 expression in tumor cells is correlated with low intratumoral infiltration of DCs and the induction of immune tolerance (Liu et al., 2016; Wang S. et al., 2022). CD47 blockade enhanced DC-mediated phagocytosis in murine colorectal cancer and reduced tumor DNA degradation in phagosomes by inhibiting phagosomal acidification (Xu et al., 2017). Tumor DNA accumulation in the cytoplasm promotes STING-dependent DNA sensing in DCs. Additionally, the CD47 blockade increased CXCL9 and IL-12 secretion by DCs to promote its intratumoral infiltration and NK cell activation (Wang S. et al., 2022). In conclusion, antagonistic monoclonal antibodies targeting CD47-SIRPα signaling are a novel immunotherapeutic strategy that functions by manipulating DNA sensing of DCs.

T-cell immunoglobulin and mucin domain containing-3 (TIM-3) is another immune checkpoint molecule exerting negative regulatory effects. TIM-3 expression on CD8^+^ T cells is a marker of T-cell dysfunction (Sakuishi et al., 2010). Indeed, DCs in tumor tissues exhibit higher TIM-3 expression than those in normal tissues, particularly cDC1s (Chiba et al., 2012; de Mingo Pulido et al., 2018). Using TIM-3 conditional knockout mice, Dixon et al. demonstrated that deletion of TIM-3 on DCs, but not on CD4^+^ or CD8^+^ T cells, promotes robust antitumor immunity (Dixon et al., 2021). TIM-3 inhibits cDC-mediated endocytosis of extracellular dsDNA, thereby preventing cytoplasmic cGAS-STING activation and IFN-I release (de Mingo Pulido et al., 2021). Additionally, DC-derived TIM-3 interacts with HMGB1 to inhibit exogenous nucleic acids from being translocated into endosomal vesicles, which limits TLR9-mediated DNA sensing (Chiba et al., 2012). Similarly, TIM-3 recruits IRF7 and p85 into lysosomes for degradation, such that it interferes with TLR9 signaling (Schwartz et al., 2017). The aforementioned studies suggest that TIM-3 on DCs is pivotal for evading nucleic acid-induced activation of the innate immune system. Thus, when TIM-3 on DCs is blocked, innate immune recognition is activated and an antitumor immune response is initiated. In a mouse breast cancer model, TIM-3 blockade effectively limits tumor growth by enhancing cGAS-STING pathway activation in CD103^+^ DCs (de Mingo Pulido et al., 2018). Following TIM-3 blockade, CXCL9 and CXCL10 expression by intratumoral DCs is high, which promotes the spatial localization of cDC1s and CD8^+^ T cells and drives the T-cell antitumor immune response in a CXCR3 receptor-dependent manner (de Mingo Pulido et al., 2018; Gardner et al., 2022). In addition, TIM-3 deletion prevents cDC1s from acquiring a regulatory program and promotes the maintenance of the CD8^+^ effector T cell pool (Dixon et al., 2021).

### Nucleic acid receptor agonist

Nucleic acid receptor agonists have unique roles in stimulating antitumor immunity. Numerous studies have used nucleic acid receptor agonists as adjuvants to increase the efficacy of cancer therapies such as chemotherapy, radiotherapy, and immune blockade therapy (Carbone et al., 2021; Lee et al., 2022). TLR9 and STING agonists targeting DNA sensors are the most advanced programs in the clinic (Table 1). By activating DNA sensors in tumor and immune cells, these agonists massively activate immune cells and increase tumor immunogenicity, which creates an altered immune landscape in the TME (Wang H. et al., 2017; Wang-Bishop et al., 2020; Cascini et al., 2023). Other strategies include using unique combinations of agonists or using the agonists as vaccine adjuvants widely available for personalized cancer immunotherapy (Kinkead et al., 2018; Ni et al., 2020; Hajiabadi et al., 2023).

**TABLE 1:** Clinical trials use agonists targeting DNA sensor for cancer immunotherapy.

Target	Drug name	Disease	Phase	ROA	Combination agent	Trial ID
TLR9	SD-101	HCC、ICC	I/II	IT	anti-PD1, anti-CTLA4	NCT05220722
		prostate cancer	II	IT	anti-PD1 and RT	NCT03007732
		pancreatic cancer	I	IT	anti-PD1	NCT05607953
		lymphoma	I	IT	anti-OX40 and RT	NCT03410901
		melanoma	I	IT	anti-PD1, anti-CTLA4, anti-LAG3	NCT04935229
TLR9	CMP-001	HNSCC	II	IT	anti-PD1	NCT04633278
		prostate cancer	II	IT	anti-PD1	NCT05445609
		breast cancer	II	IT	RT	NCT04807192
		solid tumors	II	IT	anti-PD1	NCT04916002
		lymphoma	I/II	IT	anti-PD1	NCT03983668
		melanoma	II	IT	anti-PD1	NCT04698187
		melanoma	II	IT	anti-PD1	NCT04401995
		melanoma	II/III	IT	anti-PD1	NCT04695977
		melanoma	II	IT	anti-PD1	NCT03618641
		melanoma	II	IT	anti-PD1	NCT04708418
TLR9	IMO-2125	solid tumors	II	IT	anti-PD1	NCT03865082
		solid tumors	II	IT	anti-PD1, anti-CTLA4	NCT04270864
		pancreatic cancer	I	IT	anti-PD1 and IRE	NCT04612530
		melanoma	II	IT	none	NCT04126876
TLR9	MGN-1703	melanoma	I	IT	anti-CTLA4	NCT02668770
STING	IMSA-101	solid tumors	II	IT	anti-PD1 and RT	NCT05846659
		solid tumors	I	IT	CPI	NCT06026254
		NSCLC、RCC	II	IT	CPI	NCT05846646
STING	TAK-676	solid tumors	I/II	i.v.	anti-PD1 and chemotherapy	NCT04420884
		NSCLC、TNBC、SCCHN	I	i.v.	anti-PD1 and RT	NCT04879849
STING	BI 1387446	solid tumors	I	IT	anti-PD1	NCT04147234
STING	BMS-986301	solid tumors	I	IT	anti-PD1, anti-CTLA4	NCT03956680
STING	SB 11285	solid tumors	I	i.v.	anti-PDL1	NCT04096638
STING	GSK3745417	solid tumors	I	i.v.	anti-PD1	NCT03843359
		myeloid malignancies	I	i.v.	none	NCT05424380
STING	KL340399	solid tumors	I	i.v.	none	NCT05387928
		solid tumors	I	IT	none	NCT05549804
STING	SNX281	solid tumors	I	i.v.	anti-PD1	NCT04609579
STING	CRD3874-SI	sarcoma、merkel cell cancer	I	i.v.	none	NCT06021626
STING	TAK-500	solid tumors	I/II	i.v.	anti-PD1	NCT05070247
STING	ONM-501	solid tumors	I	IT	anti-PD1	NCT06022029

Source: ClinicalTrials.gov.

HCC, hepatocellular carcinoma; ICC, intrahepatic cholangiocarcinoma; HNSCC, head and neck squamous cell carcinoma; NSCLC, non-small-cell lung cancer; RCC, renal cell carcinoma; TNBC, triple-negative breast cancer; SCCHN, squamous-cell carcinoma of the head and neck; IT, intratumoral; i.v., intravenous; ROA, route of administration; RT, radiation therapy; IRE, irreversible electroporation.

### TLR9 agonist

Currently developed TLR9 agonists are primarily synthetic oligodeoxynucleotides containing unmethylated CG dinucleotides (CpG-ODNs). They exert antitumor effects by activating pDCs and triggering interferon release. TLR9 agonists have been investigated in preclinical models as monotherapy and in combination with other cancer therapies (Carbone et al., 2021; Younes et al., 2021). Unfortunately, delivering CpG efficiently into cells has been an intractable problem. To overcome this hindrance, several preclinical studies have used liposomes as delivery vehicles or coupled TLR9 agonists to nanoparticles. These techniques increase the cycling stability of CpG while allowing for targeting cytoplasm of DCs (Kocabas et al., 2020; Du Y. et al., 2022; Sui et al., 2022). This offers a manageable delivery strategy for TLR9 agonist-based tumor immunotherapy. TLR9 agonists administered intratumorally in combination with ICIs or other immunomodulators are the latest clinical treatments. These treatments significantly improve the efficacy of tumor immunotherapy (Table 1). Intratumoral injection of SD-101, a synthetic class C CpG oligonucleotide, in combination with radiotherapy or anti-PD-1 therapy has exhibited preliminary clinical activity (Frank et al., 2018; Ribas et al., 2018; Cohen et al., 2022). Based on the compelling proof-of-concept data in mice displaying immune-mediated tumor regression, intratumoral administration of the combination of SD101 and checkpoint inhibitors is currently undergoing clinical trials for pancreatic cancer and lymphoma (Hong et al., 2022; Capacio et al., 2023). The combination of CMP-001, a virus-like particle containing the CpG-A TLR9 agonist, and anti-PD-1 has produced positive effects on PD-1-resistant advanced melanoma patients (Ribas et al., 2021). Additionally, preclinical models have suggested that intratumoral administration of CMP-001 combined with anti-PD1 therapy positively affected head and neck squamous cell carcinoma, and related clinical trials are ongoing (Cheng Y. et al., 2020). IMO-2125, an intratumoral agent, was combined with anti-CTLA4 and used against anti-PD1 refractory melanoma (Haymaker et al., 2021). Disappointingly, the results of the recent clinical phase III study on IMO-2125 unveiled that IMO-2125 combined with CTLA4 monotherapy could not achieve its objective response rate compared with CTLA4 alone. Moreover, a phase I trial of MGN-1703 combined with anti-PD-1 for advanced solid tumors is ongoing.

### STING agonist

STING agonists are used as immunotherapeutic adjuvants in numerous preclinical studies to improve the antitumor effects of tumor immunotherapy (Wang H. et al., 2017; Sun et al., 2021). The prevailing view links STING activation with increased tumor immunogenicity, immune cell activation, and initiation of antitumor-specific immunity (Woo et al., 2014; Falahat et al., 2019; Hayman et al., 2021). According to recent studies, STING plays a critical role as an inhibitor of tumor metastatic outbreaks in preventing cancer cells from progressing from a dormant state to aggressive metastasis (Hu J. et al., 2023). Notably, several human cancer types, such as colorectal cancer and melanoma, evade immune surveillance by losing STING proteins (Xia et al., 2016b; 2016a). When STING signaling is restored by epigenetic reprogramming, tumor antigenicity is improved (Falahat et al., 2023). However, STING activation also promotes tumor cell survival and drives cancer progression, metastasis, and immunosuppression (Kwon and Bakhoum, 2020). Li et al., 2022 found that STING agonists resulted in the expansion of regulatory B cells with immunosuppressive properties and inhibited NK cell-mediated antitumor immunity by secreting IL-35. STING activation not only induces immunosuppressive cell subpopulations but also upregulates the expression of immunosuppressive molecules, such as PD-L1 and IDO. This contributes to immune tolerance in cancer (Lemos et al., 2016; Luo et al., 2023). Furthermore, STING activation can induce apoptosis of DCs and T cells (Gulen et al., 2017; Pang et al., 2022). Counterintuitively, chromosomal instability (CIN)-induced chronic activation of the STING pathway in tumor cells does not trigger the interferon pathway associated with tumor immune clearance, but instead facilitates tumor immune escape through ER stress responses. In several tumor models, STING inhibitors reduce CIN-driven cancer metastasis (Li et al., 2023). In summary, STING activation plays a “double-edged sword” role in tumor immunity, and comprehending the different outcomes of STING activation can offer new ideas for STING agonist-based tumor immunotherapy.

First-generation STING agonists are derivatives of cyclic dinucleotides (CDNs). CDNs, including c-diGMP, c-diAMP, 3′,3′-cGAMP, and 2′,3′-cGAMP, are natural ligands for STING. However, CDNs application is currently limited to intratumoral administration because of its poor stability, bad cytoplasmic delivery, and insufficient activation of all human STING subtypes. Current studies on STING agonists predominantly focus on small-molecule agonists derived from non-CDNs. Through large-scale drug screening targeting the cGAS-STING pathway, researchers identified a non-nucleotide, small-molecule STING agonist, SR-717. In mouse melanoma models, intraperitoneally injected SR-717 induced adaptive immune-mediated tumor regression (Chin et al., 2020). Oral administration of MSA-2, a non-nucleotide STING agonist, augments PD-1 blockade-mediated antitumor effects in a mouse tumor model (Pan et al., 2020). Non-CDNs small-molecule STING agonists currently investigated in combination with checkpoint inhibitors are GSK3745417, KL340399, SNX281, and CRD3874-SI (Table 1). In some preclinical studies, targeted delivery of STING agonists to DCs through nanoparticles or virus-like vectors has been investigated to optimize antigen-specific antitumor immune responses (Gou et al., 2021; Jneid et al., 2023). TAK-500 is a CCR2-targeted antibody-drug coupling developed based on STING agonists and currently in clinical trials. ONM-501 is a cGAMP-loaded dual-activating polyvalent STING agonist, with the polymer used to construct micelles also binding and activating STING. Related clinical phase I trials are ongoing (Li et al., 2021).

## Concluding remarks

As innate immune cells, DCs express natural sensors to detect tumor cell-derived DNA, which initiates adaptive anti-tumor immune responses. The identification of DNA sensors, such as TLR9 and cGAS, enriched the understanding of natural immune recognition. However, there remain numerous candidate DNA sensors whose recognition mechanisms remain unclear. Multiple metabolites in the TME can modulate the DNA sensing of DCs by manipulating the metabolism of DCs. Therefore, metabolically targeted therapies against tumor cells or DCs can promote DNA sensing of DCs and require further exploration. In addition, a variety of tumor immunotherapies are designed to increase the activation of DNA sensors of DCs. A thorough understanding of the role of DNA sensors in DCs will provide insights into DC-mediated tumor immunotherapy.

## Figures and Tables

**Table:** 

**APC**	antigen-presenting cell
**AP1**	activator protein 1
**BMDC**	bone marrow-derived dendritic cell
**cDC**	conventional DC
**CTL**	cytotoxic T lymphocyte
**cGAS**	cyclic GMP–AMP synthase
**CIN**	chromosomal instability
**CDN**	cyclic dinucleotide
**CPI**	checkpoint inhibitor
**DC**	dendritic cell
**DAMP**	damage-associated molecular pattern
**DHX**	DEAH-box helicase
**DDX**	DEAD-box helicase
**DNA-PK**	DNA-dependent protein kinase
**ER**	endoplasmic reticulum
**ERAD**	endoplasmic reticulum-associated degradation
**GSDMD**	gasdermin D
**GPR81**	G protein-coupled receptor 81
**HK2**	hexokinase 2
**HSP**	heat shock protein
**IFN-I**	type 1 interferon
**IRF7**	IFN regulatory factor 7
**ICD**	immunogenic cell death
**ICI**	immune checkpoint inhibitors
**ITIM**	immunoreceptor tyrosine-based inhibitory motif
**IKK**	IκB kinase
**IRAK**	IL-1R-associated kinase
**KC**	kupffer cell
**moDC**	monocyte-derived DCs
**MyD88**	myeloid differentiation primary response protein 88
**MCT**	monocarboxylate transporter
**MV**	microvesicle
**PAMP**	pathogen-associated molecular pattern
**PRR**	pattern recognition receptor
**pDC**	plasmacytoid DCs
**PKM2**	pyruvate kinase M2
**ROS**	reactive oxygen species
**RT**	radiotherapy
**STING**	stimulator of interferon gene
**SIRPα**	signal regulatory protein α
**TLR9**	toll-like receptor 9
**TAA**	tumor-associated antigen
**TME**	tumor microenvironment
**TIR**	toll/IL-1 receptor
**TRAF6**	TNF receptor-associated factor 6
**TAK1**	TGF-β activated kinase 1
**TIM-3**	T-cell immunoglobulin and mucin domain containing-3
**TREX1**	3′ repair exonuclease 1
**UPR**	unfolded protein response
**2-DG**	2-deoxy-D-glucose
**8-OHG**	8-hydroxyguanine
